# Body mass index, sexual behaviour, and sexually transmitted infections : an analysis using the NHANES 1999–2000 data

**DOI:** 10.1186/1471-2458-6-199

**Published:** 2006-08-02

**Authors:** Nico JD Nagelkerke, Roos MD Bernsen, Sema Sgaier, Prabhat Jha

**Affiliations:** 1 Department of Community Medicine, United Arab Emirates University, Al Ain, UAE; 2 Department of Medical Microbiology, University of Manitoba, Winnipeg, Canada; 3 Centre for Global Health Research, St Michael's Hospital, Toronto, Canada

## Abstract

**Background:**

Factors determining human sexual behaviour are not completely understood, but are important in the context of sexually transmitted disease epidemiology and prevention. Being obese is commonly associated with a reduced physical attractiveness but the associations between body mass index, sexual behaviour and the risk of acquiring sexually transmitted infections has never been studied.

**Methods:**

The National Health and Nutrition Examination Survey (NHANES) files of 1999–2000 were used. Linear regression was used to relate the reported number of sex partners in the last year and lifetime to Body Mass Index (BMI). Logistic regression was used to relate Herpes Simplex Virus type II (HSV-2) antibodies to BMI and other variables.

**Results:**

Data on 979 men and 1250 women were available for analysis. Obese (mean number of partners for men:1.12, women: 0.93) and overweight (mean for men: 1.38, women: 1.03) individuals reported fewer partners than individuals of normal BMI (mean for men:2.00, women: 1.15) in the last year (p < .0.01 & p < 0.05 for men, p < 0.05 & n.s. for women). The same relationship held for lifetime partners in men (mean 11.94, 18.80, and 22.08 for obese, overweight and normal BMI respectively (p < 0.05 & n.s. for obese and overweight vs normal respectively), but not in women (mean 7.96, 4.77, and 5.24 respectively). HSV-2 antibodies were significantly correlated with the number of lifetime partners in both men and women, with the odds of being HSV-2 positive increasing by 0.6% (p < 0.01) and 2.7% (p < 0.01) for men and women respectively. HSV-2 antibodies increased with age, even after adjustment for lifetime partners (p < 0.01). Being obese (HSV-2 prevalence 15.9 and 34.9% for men and women respectively) or overweight (HSV-2 prevalence 16.7 and 29.3 for men and women respectively) was not associated with HSV-2 antibodies (HSV-2 prevalence for normal BMI: 15.6 and 23.2% respectively), independent of whether the association was adjusted for life time sexual partners or not. There was evidence of substantial misreporting of sexual behaviour.

**Conclusion:**

Obese and overweight individuals, especially men, self report fewer sex partners than individuals of normal weight, but surprisingly this is not reflected in their risk of HSV-2 infection. HSV-2 antibodies provide information not contained in self-reported number of partners and may better estimate sexual risk than self-reported behaviour.

## Background

Obesity is a risk factor for many chronic diseases, including diabetes and cardiovascular disease, and is a major causes of preventable death in Western countries, and increasingly also in developing countries[1]. In addition, obesity can also be a psychological problem as a slender posture is associated with physical and social attractiveness[2], and in most Western countries dieting, exercising etc. are multi-billion industries. Some individuals, in particular women, apparently perceive obesity as a more acute problem than cigarette smoking, which they use for weight control[3].

Despite widespread recognition that physical attractiveness correlates strongly (inversely) with body mass index (BMI), there appears to be a dearth of studies on the effectiveness of slimness in attracting sex partners, the choice of sex partners, and whether this may pose a risk for acquiring sexually transmitted infections. In addition to mere physical attractiveness psychological factors associated with BMI may also play a role in sexual behaviour.

Sexual behaviour surveys have been carried out in many countries, and their methodology has been extensively researched[4,5]. However, with rare exceptions these studies appear to have ignored the issue of body mass index as one of the few objectively measurable dimensions of physical attractiveness as a correlate of actual sexual behaviour; and those that did, appear to have included only opportunistic samples, and did not actually measure BMI[6]. Perhaps, this is because measurement of BMI involves physical examination, whereas most of these surveys are based on questionnaires or interviews.

We therefore decided to explore the relationship between obesity, sexual behaviour, and the risk of acquiring sexually transmitted infections, and estimate reported rates of partner change and the risk of Herpes Simplex Type 2 (HSV-2) antibody prevalence, a marker of cumulative sexual risk[7], as a function of body mass index, adjusting for other behavioural and demographic variables. For this, we used the (publicly available, free of charge) USA, National Health and Nutrition Examination Survey (NHANES) 1999–2000 data[8].

## Methods

### Data

The methodology of NHANES is extensively documented[8]. We downloaded and used the Demographics, Sexual Behaviour, Alcohol Use, Smoking and Tobacco Use, Body Measurement Component, Lab09 (Herpes Simplex Virus Type 1 (HSV-1) and Type-2 (HSV-2)), Laboratory 5-Urinary Chlamydia and Urinary Gonorrhoea files, and linked them using SAS 8.02 by the key variable "seqn" provided by NHANES and exported the data to SPSS (version 13.0) for subsequent analysis.

Only individuals between 20 and 49 years of age were used, as for younger individuals not all information was available. Older individuals were excluded because the relationship between current BMI and life-time number of partners or HSV-2 seropositivity could be unreliable due to the long time between many of their partnerships and BMI measurement.

In addition, all individuals reporting more same-sex (homosexual) than heterosexual life-time partners (In the NHANES survey sexual intercourse (sex) is defined as "vaginal, oral, or anal sex". Interviewing was done using the NHANES audio computer assisted self interview in either English or Spanish) were excluded as the number of individuals with predominantly same-sex partners was too small to allow a separate analysis, and we considered it incorrect to ignore the distinction between same-sex and heterosexual contacts. Also, one 29 year old woman and one 47 year old man reporting 255 and 100 partners in the last year, respectively, and an identical number of life-time partners, were excluded because the information was considered incredible.

### Statistical analyses

Standard t-tests were used for univariate analyses. We used stepwise linear regression to estimate the effects of covariables (determinants, independent variables) on life-time number of partners and number of partners during the last 12 months. The latter two dependent variables were analyzed in three different ways, *viz*. 1) directly, i.e. untransformed; 2) square root transformed; 3) logarithmically (ln(x+1)) transformed. The latter two transformations increasingly reduce the weight of individuals with many partners. The logarithmic model assumes that covariables act multiplicatively on the number of partners, while the square root of the number of partners as the dependent variable makes the model intermediate between an additive and a multiplicative one. The latter two models would seem appropriate for the number of life-time partners, as the effects of, for example, age and BMI may interact (life-time partners are accrued over time, at different rates). Both transforms also stabilize the variance in accordance with the assumptions of regression analysis. In fact, the square root transform is the variance stabilizing transformation of Poisson distributed variables and is therefore the "natural" choice for count variables[9].

Multiple logistic regression with backward selection was used to estimate the effect of covariables on the probability of having HSV-2 antibodies as a marker of cumulative sexual exposure.

No use was made of reweighting the cases using the NHANES sample weights, as we were not interested in population estimates, of – for example -prevalences, but in regression coefficients which are unbiased when the design variables of the study (mainly ethnicity and age) are included as covariables in the analysis.

Smoking was analyzed using two dummy variables, *viz*. current smoking (smoke_now) and ever smoked at least 100 life-time cigarettes (eversmoke).

For alcohol consumption we calculated the average number of weekly drinks by multiplying the number of days an individual consumes alcohol times the average number of drinks on days that individuals drank (drink_pr_wk). As we considered it unlikely that the effect of BMI on partner acquisition was linear we created two dummy variables, one (overweight) that was set = 1 for all individuals with BMI >25 and one (obese) that was set = 1 for individuals with BMI >30.

Adjustment for age always included both a linear and a square root term (ageroot) in order to avoid as much as possible any residual confounding by (a function of) age. Ethnicity was taken into account by inclusion of dummy variables for being a non-Hispanic African-American (africam), and a dummy for being a Mexican American (mexicam). All regression analyses were carried out separately for men and women as the effect of covariables may differ between the two sexes. Marital status was summarized into a single dummy variable indicating whether someone was married or living with a partner or not (partnered). Income was summarized into a dummy variable indicating an annual income of over 20,000 US $ (inc20000), as many households only reported whether their income was below or above this value. Education (1 = less than high school, 2 = high school, 3 = more than high school) was treated as a continuous variable.

In our analysis of the effect of overweight and obesity on the number of partners we treated and interpreted all other covariables as (potential) confounders. As some of these may in effect be intermediate variables, intermediate between BMI and sexual behaviour, (for example, smoking is used to lose weight) our interpretation is likely to be conservative.

## Results

A total of 979 men and 1250 women were available for analysis. Table 1 shows some of their demographic and behavioural characteristics; Table 2 shows the number of heterosexual partners in the last year and lifetime by BMI class.

Very few individuals reported any same-sex relationships. Only 29 men and 41 women reported any life-time same-sex partners, of these 20 and 34 reported <=2 such partners. Of 895 (143 HSV-2 positive) men and 1139 (333 HSV-2 positive) women the HSV-2 serostatus was known. Of 633 men and 853 women the urine Gonococcal (GC) and Chlamydia (CT) status was known. However, as only 2 men and 2 women were positive for GC and only 17 and 26 respectively for CT, we decided not to further explore these infections.

There was a large difference between the mean number of life-time sex partners reported by men, 18.18, and the number reported by women, 6.07. The same held true for the mean number of partners reported during the last year, *viz*. 1.54 and 1.03 respectively. In view of this large discrepancy between male and female number of partners, we further explored the reliability of the reported number of life-time partners by considering the HSV-2 serostatus among those individuals who reported no life-time sex partners. Of these 132 men and 152 women, 24 and 44 respectively were HSV-2 positive, substantially more than among those reporting one life-time sex partner (men:2/63, women:16/224). Although, most of these HSV-2 positive individuals probably misreported their number of life-time partners, we did not wish to exclude them to avoid introducing bias. However, as a sensitivity analysis we redid the analyses for individuals reporting at least one life-time sex partner.

### Number of partners

Our first analysis concerned the effect of BMI on the number of partners in the past 12 months. Univariately, obese (mean number of partners for men:1.12, women: 0.93) and overweight (mean for men: 1.38, women: 1.03) individuals reported fewer partners than individuals of normal BMI (mean for men:2.00, women: 1.15) in the last year (p < .0.01 & p < 0.05 for men, p < 0.05 & n.s. for women). Regressing the number of partners in the past 12 months on age, the square root of age (ageroot), African American (africam), Mexican American (mexicam), overweight, obesity, height, income (inc20000), number of drinks per week (drink_pr_wk), living with a regular partner (partnered), current smoking (smoke_now), and education, yielded 3 selected significant associations for men (Table 3), and 5 selected significant associations for women (Table 4). Using the square root and the logarithm of the number of partners in the last 12 month instead of the number itself yielded slightly different results, but was consistent as far as the effect of BMI was concerned (Tables 3 and 4). Results were largely similar when all individuals reporting no life-time sex partners were excluded (results not shown).

Subsequently, number of life-time number of partners was analyzed. Univariately, for lifetime partners the same relationship as for partners in the past 12 months held for men (mean 11.94, 18.80, and 22.08 for obese, overweight and normal BMI respectively (p < 0.05 & n.s. for obese and overweight vs normal respectively), but not for women (mean 7.96, 4.77, and 5.24 respectively). In multiple regression we regressed life time partners on the same covariables as used in the analysis of recent partners, except that instead of current smoking ever smoking was used. This yielded a significant effect of obesity on the number of reported life-time partners in men, but not in women (Tables 5 and 6), for whom the only determinant of life-time partners appeared to be cohabiting status. Using the square root and the logarithm of the number of life-time partners as dependent variable yielded some more significant associations, perhaps because of the reduced influence of outliers (Tables 5 and 6). Remarkably, obese women appeared to have a significantly higher square root number of life-time partners, but this disappeared after removal of an outlier (a woman reporting 999 life-time partners). Results were largely similar when all individuals reporting no life-time partners were excluded (results not shown).

### HSV-2 serostatus

The effect of BMI on HSV-2 serostatus was explored by regressing, using logistic regression, HSV-2 serostatus on age, the square root of age (ageroot), African American (africam), Mexican American (mexicam), overweight, obesity, height, income (inc20000), number of drinks per week (drink_pr_wk), ever smoked (eversmoke), and education; and subsequently regressing HSV-2 on the same variables plus the intermediate variables of sexual behaviour (number of life-time partners, age at first sex). Neither of these two analyses yielded a significant effect of BMI on HSV-2 serostatus (Table 7).

## Discussion

This is the first time that in a population representative sample an association between BMI and sexual behaviour has been demonstrated. The effect is substantial, with obese men reporting on average more than 10 life-time partners less than men with normal BMI. There are several possible explanations for this observation. For example, men with a desire to be highly sexual active and have many partners may invest in maintaining and cultivating an attractive body. Alternatively, obese men may not be able to attract the mates that they desire and thus have fewer partners than wanted. Also, perhaps attractive men sometimes behave opportunistically, and respond positively to women who are attracted by them.

It is interesting that BMI appears to have little effect on the self-reported number of life-time partners in women, despite its influence on recent rate of partner change.

Rather surprisingly, the number of partners in the last 12 months in men appears to be independent of age in men, while in women – not unexpectedly – it tends to decrease with age in women over 30.

The association between smoking and sexual behaviour, especially in women, which has been reported before[10,11], is interesting as it impacts on two important health-related behaviours, *viz*. smoking and obesity, and a better understanding of this relationship may be instrumental in interventions targeting high risk behaviour.

HSV-2 infection was highly prevalent in this sample, as generally in the US population. It is surprising that despite a clear influence of BMI on the number of life-time partners it appears to have little effect on HSV-2 serostatus. If HSV-2 would only be a function of the number of one's life time partners one would expect a lower HSV-2 prevalence in individuals with a high BMI. Lacking detailed data, one can only speculate why this is not the case. Perhaps, a high BMI is associated with choosing high risk partners, or with low condom use in high risk partnerships. Some of the other risk factors we identified for HSV-2 infection, such as African -American ethnicity, confirm earlier findings[12].

It is noteworthy that age – even after adjustment for the number of life-time partners – is generally associated with HSV-2 status (positively in men and young women, negatively in older women). This is in line with previous analyses of (earlier) NHANES data[13]. Perhaps, large numbers of life-time partners in young individuals reflect large numbers of partnerships of very short duration during which infection is less likely to occur. Alternatively, individuals preferentially select partners of their own age[14], and older partners are more likely to be HSV-2 positive themselves.

The association between smoking and HSV-2 serostatus has received some attention in the past[15], as has its association with other sexually transmitted infections, particularly Human Papilloma Virus, and cervical cancer[16]. In the NHANES data this association between smoking and HSV-2 disappears after adjustment for the number of life-time partners in men but not in women. Women who ever smoked were about 50% more likely to be HSV-2 infected than non-smoking women. Although, smoking may differentially affect HSV-2 susceptibility in women, residual confounding cannot be ruled out. Smoking is correlated with social-economic status (SES), and SES may be associated with variables such as choice of partner, condom use, and bias in self reported number of sex partners. Whether the same also hold true for age at first sex, which appears to be negatively associated with HSV-2 serostatus, even after adjustment for the reported number of life-time partners, is unclear. Perhaps, some biological effect that makes younger individuals more susceptible than older ones, account for this association.

Our results have to be interpreted with care. The association between BMI and the number of life time partners is (probably) diluted by the obvious fact that BMI is not constant throughout life. BMI rises with age, and although BMI at different ages is positively correlated, this correlation is by no means perfect[17]. Misreporting in sexual behaviour surveys is extremely common. For example, of the 132 men and 152 women who reported zero life-time sex partners, 24 and 44 respectively tested HSV-2 positive. Also, as is common in sexual behaviour surveys, men reported far more partners than women. This occurred despite the fact that sexual behaviour interviewing was done using the NHANES audio computer assisted self interview (in either English or Spanish) a methodology which has been widely used for eliciting information on private and sensitive issues[18].

Whether this is because women systematically under-report, or men over-report, their number of partners, or whether surveys tend to miss women with very large numbers of sex partners[19] is unclear. The hypothesis that underreporting by women rather than selection bias accounts for some of this difference is suggested by the lack of a significant age effect in the regression analyses of women's number of life-time partners. Unless younger generations are more promiscuous than older ones this would seem implausible. Interestingly, in view of the high HSV-2 prevalence among men reporting no partners, not only women appear to underreport sexual behaviour, but also some men. In view of the difficulty of obtaining reliable data on relatively simple variables such as number of partners, the task of measuring more complex behaviour such as concurrency[20], i.e. having multiple partners simultaneously instead of sequentially, is formidable and seems beyond the reach of current methods of eliciting information from informants. It emphasizes the need for better methods to measure sexual behaviour than current interviewing techniques. Perhaps, biological markers, such as antibodies to sexually transmitted infections, may provide an alternative, although – as this study also shows – we still have to learn their precise interpretation.

## Conclusion

Obese and overweight individuals, especially men, self report fewer sex partners than individuals of normal weight, but surprisingly this is not reflected in their risk of HSV-2 infection. HSV-2 antibodies provide information not contained in self-reported number of partners and may better estimate sexual risk than self-reported behaviour.

## Competing interests

The author(s) declare that they have no competing interest. No special funding was obtained for doing this study.

## Authors' contributions

The original idea is the result of extensive discussions among all authors. NN downloaded and analyzed the data. All authors contributed to the writing of the paper.

## Pre-publication history

The pre-publication history for this paper can be accessed here:



## Figures and Tables

**Table 1:** Demographic, behavioural, socio-economic, and anthropometric characteristics of sample.

**Covariable**	**Male Mean/N1/N2/N3**	**Female Mean/N1/N2/N3**
**N**	**979**	**1250**
Age (sd)	34.43 (8.49)	33.60 (8.53)
Partners last 12 months (sd)	1.54 (2.88)	1.03 (1.27)
Partners life (sd)	18.18 (57.76)	6.07 (29.14)
Age at first sex (sd)	16.39 (3.31)	17.42 (3.34)
African American (n/y)	776/203	990/260
Mexican American (n/y)	693/286	886/364
Regular Partner (n/y)	350/517	449/670
BMI (class 1/2/3)	360/363/247	434/352/452
Height (sd)	1.75 (.08)	1.62 (.07)
Income >20,000$ (n/y)	168/690	237/844
N of weekly drinks (sd)	11.30 (19.93)	6.88 (18.59)
Current smoking (n/y)	660/315	1003/246
Ever smoked (n/y)	490/486	816/433
HSV-2 antibodies (n/y)	752/143	806/333
Education (<high school/high school/high school)	330/233/414	353/300/594

**Table 2:** HSV-2 seroprevalence, age, and reported number of heterosexual partners by BMI_class.

	**Males**				**Females**			
**BMI_class**	**Last 12 months**	**Lifetime**	**HSV-2 prevalence**	**Mean Age**	**Last 12 months**	**Lifetime**	**HSV-2 Prevalence**	**Mean Age**

Normal (BMI<25)	2.00	22.08	15.6%	32.62	1.15	5.24	23.2%	32.14
Overweight (25< = BMI<30)	1.38	18.80	16.7%	35.59	1.03	4.77	29.3%	33.66
Obese (BMI>30)	1.12	11.94	15.9%	35.19	0.93	7.96^1^	34.9%	34.88

^1^.This value is heavily influenced by a single outlier reporting 999 lifetime partners. Without her this mean value goes down to 5.76.

**Table 3:** Determinants of number of partners in the past 12 months for men.

	**REGRESSION MODEL**					
	**(1) Y = N**		**(2) Y = √N**		**(3) Y = ln(N+1)**	

**Covariable**	**β**	**se(β)**	**β**	**se(β)**	**β**	**se(β)**

Age (years)	n.s.		n.s.		n.s.	
Square root of age	n.s.		n.s.		n.s.	
African American	1.058	.321	.306	.077	.233	.055
Mexican American	n.s.		n.s.		n.s.	
Regular Partner	-1.107	.253	-.214	.058	-.191	.043
Overweight	-.691	.248	n.s.		n.s.	
Obese	n.s.		-.198	.062	-.148	.046
Height	n.s.		n.s.		n.s.	
Income >20,000$	n.s.		n.s.		n.s.	
N of weekly drinks	n.s.		n.s.		n.s.	
Current smoking	n.s.		n.s.		n.s.	
Education	n.s.		n.s.		n.s.	
Constant	2.684	.249	1.237	.052	.923	.038

n.s. = not selected by stepwise regression

Stepwise regression (p entry .05, p removal .10) of number of partners in the last 12 months, and the root number of partners in the last 12 months on selected covariables. The β coefficients represent the increase in the outcome (dependent) variable (e.g. number of partners N) for every unit increase (e.g. one year, for age) of the covariable to which it belongs. Thus, regression model (1) below can be expressed as N = 1.058.I(African American) - 1.107.I(has a regular partner) -0.691.I(BMI>=25)+2.684, where I(condition) = 1 if the individual meets that condition (e.g. the individual is an African American) and = 0 otherwise.

**Table 4:** Determinants of number of partners in the past 12 months for women.

	**REGRESSION MODEL**					
	**(1) Y = N**		**(2) Y = √N**		**(3) Y = ln(N+1)**	

**Covariable**	**β**	**se(β)**	**β**	**se(β)**	**β**	**se(β)**

Age (years)	-.012	.004	-.097	.034	-.067	.026
Square root of age	n.s.		1.029	.392	.694	.305
African American	.180	.091	n.s.		.066	.033
Mexican American	n.s.		n.s.		n.s.	
Regular Partner	-.172	.074	n.s.		n.s.	
Overweight	-.221	.072	-.094	.034	-.082	.027
Obese	n.s.		n.s.		n.s.	
Height	n.s.		n.s.		n.s.	
Income >20,000$	n.s.		n.s.		n.s.	
N of weekly drinks	n.s.		n.s.		n.s.	
Current smoking	.334	.082	.117	.039	.101	.030
Education	n.s.		n.s.		n.s.	n.s.
Constant	1.694	.153	-1.654	1.117	-1.037	.870

n.s. = not selected by stepwise regression

Stepwise regression (p entry .05, p removal .10) of number of partners in the last 12 months, and the root number of partners in the last 12 months on selected covariables. The β coefficients represent the increase in the outcome (dependent) variable (e.g. number of partners N) for every unit increase (e.g. one year, for age) of the covariable to which it belongs.

**Table 5:** Determinants of life time number of partners for men.

	**REGRESSION MODEL**					
	**(1) Y = N**		**(2) Y = √N**		**(3) Y = ln(N+1)**	

**Covariable**	**β**	**se(β)**	**β**	**se(β)**	**β**	**se(β)**

Age (years)	.906	.292	n.s.		-.184	.089
Square root of age	n.s.		.845	.161	2.482	1.028
African American	26.72	6.611	1.197	.316	.482	.125
Mexican American	n.s.		-.799	.253	-.459	.100
Regular Partner	n.s.		-.504	.253	-.271	.102
Overweight	n.s.		n.s.		n.s.	
Obese	-13.22	5.61	-.748	.253	-.292	.100
Height	n.s.		n.s.		n.s.	
Income >20,000$	n.s.		-.759	.283	-.329	.113
N of weekly drinks	n.s.		n.s.		n.s.	
Smoking (ever)	n.s.		n.s.		.189	.089
Education	n.s.		n.s.		n.s.	
Constant	-12.65	10.44	-.446	.936	-5.478	2.913

n.s. = not selected by stepwise regression

Stepwise regression (p entry .05, p removal .10) of number of number of life time partners, and the root number of life time partners on selected covariables. The β coefficients represent the increase in the outcome variable (e.g. number of partners) for every unit increase (e.g. one year, for age) of the covariable to which it belongs.

**Table 6:** Determinants of life time number of partners for women.

	**REGRESSION MODEL**					
	**(1) Y = N**		**(2) Y = √N**		**(3) Y = ln(N+1)**	

**Covariable**	**β**	**se(β)**	**β**	**se(β)**	**β**	**se(β)**

Age (years)	-.012	.004	-.097	.034	-.067	.026
Square root of age	n.s.		1.029	.392	.694	.305
African American	.180	.091	n.s.		.066	.033
Mexican American	n.s.		n.s.		n.s.	
Regular Partner	-.172	.074	n.s.		n.s.	
Overweight	-.221	.072	-.094	.034	-.082	.027
Obese	n.s.		n.s.		n.s.	
Height	n.s.		n.s.		n.s.	
Income >20,000$	n.s.		n.s.		n.s.	
N of weekly drinks	n.s.		n.s.		n.s.	
Current smoking	.334	.082	.117	.039	.101	.030
Education	n.s.		n.s.		n.s.	n.s.
Constant	1.694	.153	-1.654	1.117	-1.037	.870

n.s. = not selected by stepwise regression

Stepwise regression (p entry .05, p removal .10) of number of number of life time partners, and the root number of life time partners on selected covariables. The β coefficients represent the increase in the outcome variable (e.g. number of partners) for every unit increase (e.g. one year, for age) of the covariable to which it belongs.

**Table 7:** Determinants of HSV-2 serostatus.

	**Male**				**Female**			
	
	**Not adjusted for life time partners**		**Adjusted for life time partners +age at first sex**		**Not adjusted for life time partners**		**Adjusted for life time partners+age at first sex**	
**Covariable**	**β**	**se(β)**	**β**	**se(β)**	**β**	**se(β)**	**β**	**se(β)**

Age (years)	n.s.		n.s.		-.931	.224	-.883	.231
Square root of age	.821	.175	.785	.184	11.47	2.64	10.96	2.72
African American	1.396	.248	1.044	.265	1.796	.230	1.723	.236
Mexican American	n.s.		n.s.		-.648	.246	-.497	.257
Overweight	n.s.		n.s.		n.s.		n.s.	
Obese	n.s.		n.s		n.s.		n.s.	
Height	n.s.		n.s.		n.s.		n.s.	
Income >20,000$	n.s.		n.s.		-.933	.233	-.862	.245
N of weekly drinks	n.s.		n.s.		n.s.		n.s.	
Smoking (ever)	.402	.234	n.s.		.586	.185	.419	.195
N lifetime partners			.006	.002			.027	.011
Age at first sex			-.075	.037			-.060	.030
Education	n.s.		n.s.		n.s.		n.s.	
Constant	-7.246	1.081	-5.65	1.26	-35.45	7.68	33.32	7.92

n.s. = not selected by stepwise regression

Stepwise regression (backward selection) of HSV-2 serostatus on selected covariables. For dichotomous covariables the β coefficients are the natural logarithms of the adjusted odds ratios of the association between the covariable and HSV-2 seropositivity. Analyses adjusting for age at first sex (firstsex) as a covariable exclude individuals without sex partners.
